# Pathway analysis comparison using Crohn's disease genome wide association studies

**DOI:** 10.1186/1755-8794-3-25

**Published:** 2010-06-28

**Authors:** David Ballard, Clara Abraham, Judy Cho, Hongyu Zhao

**Affiliations:** 1 Robert S. Boas Center for Human Genetics and Genomics, Feinstein Institute for Medical Research, Manhasset, New York, USA; 2 IBD Center, Division of Gastroenterology, Department of Medicine, Yale University, New Haven, CT, USA; 3 Department of Genetics, Yale University, New Haven, CT, USA; 4 Department of Epidemiology and Public Health, Yale University, New Haven, CT, USA

## Abstract

**Background:**

The use of biological annotation such as genes and pathways in the analysis of gene expression data has aided the identification of genes for follow-up studies and suggested functional information to uncharacterized genes. Several studies have applied similar methods to genome wide association studies and identified a number of disease related pathways. However, many questions remain on how to best approach this problem, such as whether there is a need to obtain a score to summarize association evidence at the gene level, and whether a pathway, dominated by just a few highly significant genes, is of interest.

**Methods:**

We evaluated the performance of two pathway-based methods (Random Set, and Binomial approximation to the hypergeometric test) based on their applications to three data sets of Crohn's disease. We consider both the disease status as a phenotype as well as the residuals after conditioning on IL23R, a known Crohn's related gene, as a phenotype.

**Results:**

Our results show that Random Set method has the most power to identify disease related pathways. We confirm previously reported disease related pathways and provide evidence for IL-2 Receptor Beta Chain in T cell Activation and IL-9 signaling as Crohn's disease associated pathways.

**Conclusions:**

Our results highlight the need to apply powerful gene score methods prior to pathway enrichment tests, and that controlling for genes that attain genome wide significance enable further biological insight.

## Background

In order to increase the power of genome wide association studies (GWAS) to identify the biological mechanisms underlying complex disease, the field is leveraging many methodological advances on the analysis of gene expression microarray data. In a standard gene expression analysis, measured probe intensities are first summarized to estimate the expression level of a gene, and these genes are often ranked according to the statistical evidence, such as derived from T or moderated T-tests, of their differential expression between conditions. The genes at the top of the list are then considered for follow-up studies, but it is a nontrivial decision on how many of these genes to pursue for further investigation. Biological annotation is often incorporated to facilitate this decision. It is unclear that using annotation information in the analysis of GWAS data would attain the same increase in power seen in gene expression analysis because functional and genetic variation have not been found to exhibit the same clustering within pathways as gene expression [1]. However, Elbers et al. [2] conclude that disease related genes may be functionally related and grouped in pathways due to the small number of pathways that are assumed to contribute to the development of complex diseases. Additionally, several studies have reported pathway analysis results for GWAS [3-6] showing the power of this approach to identify disease associated pathways.

There are a number of statistical issues to address in implementing pathway analysis. First and foremost, a decision has to be made on which collection of pathways to consider and which gene annotation to use in the analysis. Second, the boundary regions need to be defined around a gene. Third, a statistical method needs to be selected 1) to compute a gene level measure of association to the disease status (a gene score) if such a gene-based summary is needed, and 2) to identify pathways either enriched for association signals or highly predictive of disease status. Additional issues involve handling genes in linkage disequilibrium and genes residing in multiple pathways.

Several association tests can be used to calculate gene level scores. The most commonly used method is to identify the SNP with the lowest p-value and then allow for multiple comparisons through permutation, however, it is not the most powerful [7,8]. Our simulation study has shown that PCA regression is more powerful than the best scoring SNP when including the possibility of multiple causal SNPs of varying allele frequencies [8]. These gene scores are then used to determine which pathways are enriched in the data set. One method to determine pathway enrichment is with contingency table tests: chi-square test, Fisher's Exact test, or a binomial test [see 9 for a review]. Another method is described by Newton et al. [10], it incorporates the use of random-set methods to determine the enrichment of genes in a pathway. These methods use the same null hypothesis as the contingency table methods above, but determine their significance by creating an empirical distribution of a pathway's significance by using random sets of genes. (Please see Cantor et al. [11] for a review of statistical methods.)

Here we use several analysis methods on two well publicized datasets of Crohn's disease. We apply two methods to compute gene level scores as well as two different methods to identify pathways associated with the disease. We also investigate the impact of IL23R to the pathway results by two methods. First, we remove IL23R from the list of measured genes and rerun the analysis. Second, we condition the disease status on IL23R and use the residuals as the outcome variable. IL23R was first identified and replicated in the datasets used in our analysis and is the highest ranking known gene in each dataset. By removing IL23R, we can evaluate each method's ability to identify disease related pathways after the removal of major genetic contributors. Lastly, we explore our pathway results to identify other genes implicated in the disease.

## Results

### Gene Scores

The top ten genes for each dataset using PCA regression contain known Crohn's related genes. In the Jewish (J) dataset IL23R, PHOX2B, IL13, and MAST3 were ranked 3rd, 4th, 8th, and 9th, respectively (Table 1). IL23R and NOD2 were ranked first and second, respectively in the non-Jewish (NJ) dataset (Table 1). And, IL23R, IRGM, NOD2, PTPN2 were ranked 2nd, 4th, 7th and 10th in the Wellcome Trust (WTCCC) dataset (Table 1). Interestingly, after variation explained by IL23R was removed from the data and gene scores were recalculated, the top genes remained consistent. Note that for the pathway enrichment analysis, all genes with SNPs mapped within their boundaries were used.

**Table 1:** Top 10 ranking genes for each dataset using conditioned and unconditioned data

Dataset	Unconditioned	Conditioned
Jewish	ZNF282	ZNF282

	ZNF398	ZNF398

	IL23R	MRPL48

	PHOX2B	TMEM183A

	MRPL48	FLJ20366

	BRD7	FOXD3

	FLJ20366	EBAG9

	IL13	PHOX2B

	MAST3	LOC441843

	IL4	TSC22D1

Non-Jewish	IL23R	CYLD

	NOD2	SGCEP

	CYLD	NOD2

	SGCEP	NAT2

	NAT2	SCARNA5

	SCARNA5	ZDHHC4

	SCL9A5	C7orf26

	VPS4A	PPP2R5E

	FLJ35695	FLJ35695

	SSH2	DEFB121

WTCCC	ZNF135	ZNF135

	IL23R	DGKD

	DGKD	ADIG

	IRGM	IRGM

	ADIG	CYLD

	CYLD	LOC441108

	NOD2	ARNT

	C1orf141	NOD2

	ARNT	PTPN2

	PTPN2	PSMD5

### Significant Pathways

The RS method applied to gene scores calculated from PCA regression identified 38, 14, and 7 pathways as significant for the WTCCC, J, and NJ datasets, respectively. Two pathways were common to all three datasets, ten were in common only between the WTCCC and J datasets, one between only WTCCC and NJ. The pathways in common across all datasets are BioCarta's IL-2 Receptor Beta Chain in T Cell Activation Pathway and GenMAPP's IL-9 signaling pathway (Table 2). All significant pathways are shown in Additional file 1 Table S1. Genes for each pathway are listed in the Additional file 2 Tables S3 and S4. Applying the BIN enrichment method to the same data, 36 pathways are significant for WTCCC, 6 for J, and 2 for the NJ. Of the 36 pathways in the WTCCC, 27 are in common to those identified by RS, 4 of the 6 for the J and zero of the NJ are in common. In the J dataset, the adipogenesis pathway as well as IL-22, -7 and NF-κB pathways were identified. Pathways found using the BIN method are listed in Additional file 3 Table S2.

**Table 2:** Summary of significant pathways identified

Pathway	Method	IL23R status	Dataset
IL-9 Signaling	RS	in	WTCCC, J, NJ

IL-2 Receptor Beta Chain in T cell Activation	RS	in	WTCCC, J, NJ

Activation of Csk by cAMP-dependent Protein Kinase Inhibits Signaling through the T Cell Receptor pathway	RS,BIN	in, out, cond	WTCCC

Ahr Signal Transduction Pathway	RS	in, out, cond	WTCCC

Antigen Dependent B Cell Activation pathway	RS	in, out, cond	WTCCC

Bystander B Cell Activation pathway	RS	in, out, cond	WTCCC

Cytokines and Inflammatory Response pathway	RS, BIN	in, out, cond	WTCCC

Erythropoietin mediated neuroprotection through NF-kB pathway	RS	in, out, cond	WTCCC

Hypoxia-Inducible Factor in the Cardiovascular System pathway	RS	in, out, cond	WTCCC

IL 4 signaling pathway	RS	in, out, cond	WTCCC

IL 5 Signaling Pathway	RS	in, out, cond	WTCCC

IL12 and Stat4 Dependent Signaling Pathway in Th1 Development pathway	RS	in, out, cond	WTCCC

IL22 Soluble Receptor Signaling Pathway	RS	in, out, cond	WTCCC

Lck and Fyn tyrosine kinases in initiation of TCR Activation pathway	RS, BIN	in, out, cond	WTCCC

NO2-dependent IL 12 Pathway in NK cells pathway	RS, BIN	in, out, cond	WTCCC

Selective expression of chemokine receptors during T-cell polarization pathway	RS	in, out, cond	WTCCC

Glycerolipid metabolism	RS	in, out, cond	WTCCC

Glycerophospholipid metabolism	RS	in, out, cond	WTCCC

Adipogenesis Human	RS, BIN	in, out, cond	WTCCC

IL-4 signaling pathway	RS, BIN	in, out, cond	WTCCC

Kit Receptor Signaling Pathway	RS, BIN	in, out, cond	WTCCC

TPO Signaling Pathway	RS	in, out, cond	WTCCC

Role of Erk5 in Neuronal Survival pathway	BIN	in, out, cond	WTCCC

Transcription factor CREB and its extracellular signals pathway	BIN	in, out, cond	WTCCC

Acetylcholine Synthesis	BIN	in, out, cond	WTCCC

Glycosphingolipid biosynthesis	RS	in, out, cond	J

Deregulation of CDK5 in Alzheimers Disease pathway	RS	in, out, cond	NJ

The table summarizes the significant pathways identified in the three datasets, Wellcome trust Case-Control Consortium (WTCCC), Jewish (J), non-Jewish (NJ), that were significant using either method, Random Set (RS) or Binomial (BIN). The table also notes how IL23R was treated in the enrichment analysis, included (in), excluded (out), or conditioned upon (cond). The complete list of significant pathways are found in Additional Tables 1 and 3.

The same enrichment methods were applied after disease status had been conditioned on IL23R. In the WTCCC dataset 32 pathways were significant by the RS method, 21 of the 32 were in common to the significant pathways above which included IL23R. The BIN method determined 17 pathways significant, 10 of which were in common to those identified when IL23R was included. Removing the variation of IL23R had a larger impact on the J and NJ datasets than WTCCC. Using RS, only three pathways were significant in the J population, one of which was significant previously. The BIN method determined two as significant, none matching the unconditioned analysis. In the NJ data, the RS method found four pathways significant, none in common to pathways when IL23R variation was included. The BIN method found no pathways significant following the removal of IL23R. There were no common pathways across the three datasets using either RS or BIN; however, BioCarta's 'Cells and Molecules Involved in Local Acute Inflammatory Response' was significant in both the J and NJ datasets with RS. See Additional file 1 Table S1.

**Figure 1:**
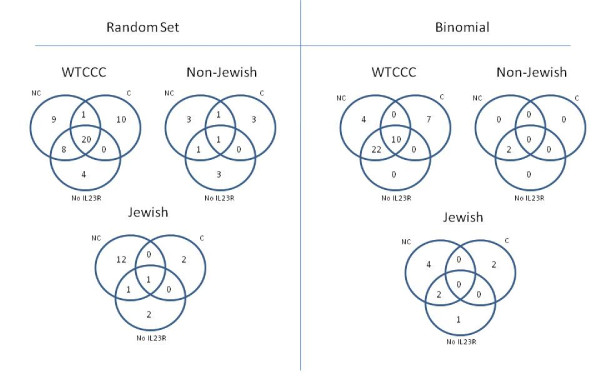
**Overlap of significant pathways identified by each method (Random Set, Binomial) in each of the datasets (WTCCC, Non-Jewish, Jewish)**. Numbers are equal to number of significant pathways for each analysis: NC = non-conditioned, C = conditioned, NoIL23R = IL23R removed from gene list.

If IL23R is removed from the list of measured genes and the analysis is performed, the significant pathways are generally the same as those when IL23R is included. Using RS, 32 pathways are significant in WTCCC, 28 of which overlap with pathways identified when IL23R is included; six are significant in J, two overlapping those including IL23R; and five in the NJ where two overlap significant pathways found using IL23R. The BIN results were more consistent to pathways identified using IL23R. Figure 1 shows the overlap for each method.

### IL23R Effect

IL23R is the highest ranked known Crohn's disease gene in each of the data sets and it is present in 29 of the pathways used in our analysis. In fact for the RS results, 27 of the 38 WTCCC significant pathways, 12 of the 14 significant J pathways, and three of the seven significant NJ pathways contain IL23R. Using BIN the influence of IL23R is reduced; nevertheless, 26 of 36 in WTCCC and four of six in J significant pathways contained IL23R. Given that there is overlap in the pathways identified as significant between the conditioned and unconditioned and removal of IL23R data (10 in the WTCCC using RS, 4 using BIN) IL23R is not responsible for making all of the IL23R containing pathways significant. The impact on the J and NJ data is more dramatic; all IL23R containing pathways are no longer significant. When IL23R is simply removed from the analysis a similar decrease in the number of identified pathways decreases in the J and NJ.

Given the lack of fluctuation in gene scores following conditioning in the WTCCC data, we were curious to see eleven new pathways reaching significance in the conditioned data by RS (Table 2 and Additional file 1 Table S1). Twenty-one of the 32 significant pathways were in common to the unconditioned data and represent expected Crohn's disease pathways: interleukin signaling, B Cell, and cytokine signaling pathways. The other eleven pathways are pathways not previously identified in other pathway analyses of Crohn's disease. Looking at the genes that make up these pathways, many are found in more than one pathway. For example, Wnt signaling genes (WNT3, WNT3A, WNT9A, WNT9B with p-values 1.8 × 10^-3^, 3.6 × 10^-3^, 1.2 × 10^-3^, and 6.8 × 10^-5^, respectively) and calcium/calmodulin dependent protein CAMK2G (p-value 2.0 × 10^-3^) are in four pathways. Interestingly, the WNT genes above had comparable gene score before and after conditioning; however, the effect of IL23R was able to mask their association to Crohn's disease.

When IL23R is removed from the analysis, the number of significant pathways decreases using both RS and BIN. Using RS, the significant pathways identified for WTCCC are by and large the same significant pathways when IL23R is included in the analysis. These pathways were primarily made of up cytokine signaling, T-cell, and B-cell pathways. In the J dataset, only two pathway out of four overlaps those found using IL23R. All of the pathways identified were cytokine pathways. The only pathway identified by all three methods was 'Glycosphingolipid biosynthesis'. The NJ data had two out of five significant pathways that overlapped pathways found when IL23R was included. 'Deregulation of CDK5 in Alzheimer's Disease pathway' was significant in each method (Table 2). The BIN results identified few pathways as well. All identified pathways in the WTCCC and NJ data overlapped pathways identified using IL23R, the J identified one pathway, 'Inflammatory Response', that did not overlap.

### Choice of Gene Score Method

The number of significant pathways identified using the pathway enrichment methods is larger when the gene score is calculated with PCA regression compared to the best scoring SNP within the gene. Table 3 lists the number of pathways identified as significant for the three data sets using the BI method on gene scores calculated by using the normalized, most significant SNP within the gene as the gene score. This method is the most commonly used method for calculating gene scores from GWAS data. Only five, zero, and two pathways were enriched using this gene score for the WTCCC, J, and NJ datasets, respectively. This is compared to 36, 6, and 2 (WTCCC, J, NJ) when the gene scores are calculated using PCA regression.

**Table 3:** Number of significant pathways identified using PCA regression and Best scoring SNP within a gene to calculate gene level scores

Pathway method	Data set	PCA	Best Scoring SNP
Binomial	Jewish	6	0

	Non-Jewish	2	2

	WTCCC	36	5

## Discussion

Over the past few years, GWAS studies have had a large impact on identifying the determinants of complex diseases, and Crohn's disease has been the focus of many researchers. Here we used two studies, both of which implicated novel susceptibility genes for Crohn's disease, as a basis for identifying biological pathways associated with the disease. We applied two methods to calculate gene score association, and three analyses to determine pathway enrichment. We also evaluated the impact that a highly associated known disease related gene has on the identification of enriched pathways.

Previous analyses of Crohn's disease describe an enrichment of pathways involved in calcium signaling, Transcription/ChREBP regulation, Immune response (IL2, IL3, IFN alpha/beta, antigen presentation), lipid metabolism, and the developmental role of CDK5 in neuronal development [5]. Wang et al. [6] identified enrichment of the IL12 signaling pathway in multiple data sets, and Holmans et al. [12] found enrichment of the major histocompatibility complex (MHC), immunological response, and antigen processing gene ontology categories. These results were found using the same WTCCC data set analyzed here. Despite the differences in implementation across methods (e.g., how SNPs are assigned to genes, definition of gene boundaries, gene set annotation used, pathway enrichment determination) there are common themes to the results. The main commonality is the immune response (interleukin) pathways which represent the immune system's complex signaling response to microbial infection and injury, as well as the dysregulated response observed in disease. These are perhaps the most expected given the association of IL23R and other pathways ultimately affecting interleukin signaling to Crohn's disease [13,14]. Our results confirmed the significance of many of the immune response pathways, and although IL12 was not our highest ranking pathway as in Wang et al. [15]; it was found to be significant. Additionally, of the potential Crohn's disease susceptibility genes suggested by Wang et al. [15], only IL12B obtained a low gene score (4.67 × 10^-3^) in our analysis. In our annotation of IL12 pathways, IFNGR1 had the most significant gene score (6.82 × 10^-7 ^unconditioned, 5.10 × 10^-6 ^after conditioning). We also confirmed the association of lipid/adipogenesis pathways and CDK5 first proposed by Torkamani et al. (2007). Our CDK5 enrichment was found in the non-Jewish dataset, not in the WTCCC. Calcium signaling pathways proposed by Torkamami et al. [5] were not significant in our analysis. However, RHOA was responsible for pushing several pathways to significance in the WTCCC data. This difference may be due to a difference in pathway definition. Several MHC genes were identified as significant in our analysis. Holmes et al. [12], who looked at the over-representation of GO categories, detailed several genes in their enriched MHC Go category as potential Crohn's genes. Our analysis found HLA-DQA2, HLA-DQB2, HLA-DOB, and HLA-DRA, to be highly associated with the disease. We did not, however, have an MHC pathway in our annotation. Lastly, we identify many of the same pathways as significant as Peng et al.[4]: IL -2, -3, -4, -6, -12 signaling, Sphingolipid Metabolism, CDK5, T Cell Receptor, Cytokine and Inflammation related pathways. In their study, they computed gene scores using the best scoring SNP within a gene and used Fisher Exact Test and Simes/FDR to identify significant pathways in several datasets, including WTCCC. Like our results, the Fisher Exact Test was the more conservative of the methods used.

Our results identified two pathways (IL-2 Receptor Beta Chain in T cell Activation, IL-9 Signaling) as significant in each dataset with RS, which weights genes by their p-value. These findings are consistent with known functional roles of these pathways in intestinal immune homeostasis. For example, the IL-2 pathway is critical in T cell activation and regulatory T cell function. As such, IL-2R-deficient animals develop intestinal inflammation [16]. Moreover, the IL-9 signaling pathway enhances regulatory T cell function, Th17 and Th2 differentiation [17,18], as well as regulating mast cell and goblet cell function [19-21], all of which are critical in intestinal immunity. Using BIN, they are only significant in the WTCCC data. This is likely due to the increase in power associated with the larger sample size in the WTCCC dataset compared to the J and NJ datasets. Once variation from IL23R is removed, these two pathways are no longer significant in any of the datasets. Interestingly, our 'Autophagy' pathway based on Refseq gene descriptions was not significant in any dataset using either method.

Our earlier simulation study showed that PCA regression had more power to identify associations than using the best scoring SNP within a gene when the association was due to multiple SNPs within a gene. We created gene scores using each method and applied our binomial test to each of dataset using a p-value threshold of 0.05. The comparable number of identified known genes using a liberal threshold may not be unexpected given that these genes were originally identified using a single SNP analysis. However, using these gene scores to perform pathway analysis resulted in quite different results - PCA gene scores identified more disease related pathways. Given the success of PCA applied to genes within a gene, we investigated using PCA applied to all SNP within all genes of a pathway and then performing the regression. The results did not identify any enriched pathways.

The difference in the number of significant pathways identified between the RS and BIN methods illustrates the impact that highly significant genes within a pathway can have on pushing a pathway to becoming significant. Weighting genes by their significance may increase power to identify pathways, but highly significant genes would have been found with a single SNP analysis and their annotated pathways would have warranted follow-up without a pathway analyses. Given that IL23R was significant in each of the datasets, we evaluated the impact of IL23R on the resulting pathway analysis. Removing this variation had a large impact on the smaller J and NJ datasets; it resulted in removing all interleukin-related pathways from significance. There were, however, a few remaining pathways that maintained their significance and contain genes that have an implicated role in Crohn's disease. The ability of the Jewish population to maintain some power compared to the non-Jewish population could be explained by the higher prevalence of Crohn's disease observed in the Ashkenzi Jewish population compared to similar non-Jewish populations. The WTCCC dataset was significantly larger than either the J or NJ datasets, and provided more power to identify disease related pathways. Even when conditioned on IL23R, many interleukin pathways remained significant. Of interest, though, is that removing IL23R variation allowed other pathways to become significant that were not previously: two different annotations of WNT signaling pathways became significant. These results match previously published IBD gene expression studies which found significantly increased expression of seven WNT genes in IBD patients compared to non-IBD controls [22].

## Conclusions

While many issues regarding pathway analysis in GWAS remain (which annotation collection is more appropriate, how many base pairs around a gene should be included to account for linkage disequilibrium when calculating gene scores), its application has identified appropriate disease related pathways in multiple studies. Here, we show the impact of using a less powerful gene score method as input to gene set enrichment in GWAS as well as the effect a single highly significant gene can have on the identified pathways. Pathway enrichment methods that do not incorporate a gene score, however, may be less biased towards pathways that contain genes near highly significant SNPs. Importantly, regardless of the pathway method used, removing the variation explained by significant genes did not eliminate the ability of pathway enrichment methods to identify disease related genes.

## Methods

We consider three Crohn's disease data sets obtained from two previously published studies. Duerr et al.[13] performed the first case-control GWAS for inflammatory bowel disease. This study included two subject cohorts, Jewish and non-Jewish. The non-Jewish cohort consisted of 567 cases and 571 controls; whereas the Jewish cohort included 401 cases and 433 controls. Each subject was genotyped using Illumina's HumanHap300 BeadChip. This study identified a number of independent associations in the IL23R gene region for both Crohn's disease and ulcerative colitis, in addition to confirmation of the previously identified Crohn's disease associations with NOD2. Following this finding, the Wellcome Trust Case Control Consortium [23] published GWAS results of seven complex diseases including Crohn's disease. For each disease, the Consortium enrolled 2,000 subjects with the disease but used a common set of 3,000 controls to test for association. Their control group contained 1,500 individuals from two sources - the 1958 British Birth Cohort and blood donors from the UK blood Services. All subjects in this study were genotyped using the Affymetrix GeneChip Human Mapping 500 K Array Set. We apply each method to Duerr et al's Jewish (J), non-Jewish (NJ), and Welcome Trust Case Control Consortium's (WTCCC) inflammatory bowel/Crohn's disease data sets.

We included only SNPs with a missing rate <10%, that were in Hardy Weinberg equilibrium (chi-square p-value greater than 0.001), and had a minor allele frequency greater than 1% in each data set. Subjects with greater than 6% missing values were removed from the analysis. The SNP were mapped to known genes using Refseq annotation downloaded from NCBI http://www.ncbi.nlm.nih.gov/. A SNP was considered 'in' a gene if it was located within the gene boundaries plus/minus 10 kb. There were SNPs that did not get mapped to a gene in each dataset. Our pathway annotation was compiled from three public databases: KEGG [24], GenMAPP [25], and BioCarta [26]. We created one additional pathway by searching Refseq annotation gene descriptions for the word 'autophagy'. Such a pathway is of interest because autophagy genes have been implicated in Crohn's disease [27]. There were a total of 565 pathways consisting of a total of 5,464 unique genes; note that one gene may occur in more than one pathway. The median number of genes per pathway is 28 genes, and pathways averaged 47 genes in size.

We calculated each gene level score using SNPs mapped to genes, where the SNPs were converted from character (A, T, C, G) into the count of the minor allele. Principal component analysis (PCA) was applied to the correlation matrix of the gene's SNPs, and then the components that explained at least 85% of the variation were used as explanatory variables in the regression to explain disease status. The p-value from this multiple regression is the gene score. For comparison purposes, gene level scores using the SNP with the lowest p-value within the gene boundaries were calculated as well. Permutation of the case control status was used to standardize the p-value within each gene.

We identified disease associated pathways using two methods. The first method was the binomial test (BIN), an approximation to the Fisher's Exact Test. The binomial approximation has been widely used in gene expression studies [28] and is computationally faster than Fisher's Exact Test. Genes were significant if their gene scores were less than 0.05. The test statistic of our second method, the random set method (RS), weighs each gene by its p-value to determine enrichment. Specifically, our RS method's test statistic is equal to the negative of the sum of the log p-values for each gene assigned to the pathway [10]. An approximate FDR was calculated for each method using a two step procedure. Step one - gene scores were computed using 1000 permutations of disease status. Step two - for each set of permuted gene scores, pathway enrichment was calculated using 10,000 permutations of the gene scores. The approximate false discovery rate was calculated by averaging the number of pathways with a p-value less than that obtained using real data. Pathways with an estimated FDR less than 0.01 are reported.

We evaluate the effect of a highly significant gene found in each of the data sets (IL23R) by regressing case control status onto SNPs located within IL23R. The residuals from the regression were then used as the outcome variable and PCA regression was used to recalculate all of the gene scores. These gene scores and permutations of the residuals were used in the same way described above to calculate and estimated FDR for each method. The accuracy was assessed using the mean square error and permutation was used to obtain the alpha 0.01 cut-off.

## Competing interests

The authors declare that they have no competing interests.

## Authors' contributions

DB carried out the computational and statistical analysis. CA and JC provided immunological interpretation to the results. JC provided the J and NJ data sets. HZ participated in study design and guidance in analysis. All authors approved the manuscript.

## Pre-publication history

The pre-publication history for this paper can be accessed here:

http://www.biomedcentral.com/1755-8794/3/25/prepub

## Supplementary Material

Additional file 1 **Significant pathways from Random Set analysis**. The table lists the significant pathways across dataset (WTCCC, Jewish, non-Jewish) and IL23R status (included, excluded, conditioned upon). An 'X' means the pathway had an FDR less than 0.01. Click here for file

Additional file 2 **Genes located in significant pathways across three datasets**. The table lists the genes located in the two pathways 'IL-2' and 'IL-9' which were significant in each dataset evaluated. across dataset (WTCCC, Jewish, non-Jewish) and IL23R status (included, excluded, conditioned upon). An 'X' means the pathway had an FDR less than 0.01. Each gene is followed by the p-value obtained from PCA regression of disease status onto SNPs located within the gene. Click here for file

Additional file 3 **Significant pathways from Binomial analysis**. The table lists the significant pathways across dataset (WTCCC, Jewish, non-Jewish) and IL23R status (included, excluded, conditioned upon). An 'X' means the pathway had an FDR less than 0.01. Click here for file
